# Push-pull-mooring model-based study of medical personnel’s shift intention to rural areas and the quality of public health services: a test of empirical data in Zhejiang Province

**DOI:** 10.3389/fpubh.2025.1647150

**Published:** 2025-09-30

**Authors:** Wu Li, Zheng Yuan, Xu Jiayi, Huo Rongmian

**Affiliations:** College of Humanities and Foreign Languages, China Jiliang University, Hangzhou, China

**Keywords:** medical personnel, structural equation models, medical service quality, shift intention to rural areas, push-pull-mooring model

## Abstract

**Background:**

Aiming at the current situation of shortage of medical personnel in rural areas of China, combined with the gap between primary health and preventive services and the health needs of the public, we explore the influencing factors of medical personnel’s shift intention to rural areas, and propose countermeasures to improve the quality of rural medical services and the medical security system.

**Subjects and methods:**

Based on the push-pull-mooring model, medical professionals and medical students within five prefectural-level cities (Hangzhou, Jinhua, Wenzhou, Quzhou, and Lishui) in Zhejiang Province were selected for a survey, and a model of medical personnel’s intention to shift to rural areas was established and validated using the Amos (CB-SEM), which examined the influence of healthcare resource supply, public healthcare quality, and social support on the medical personnel’s shift decision-making.

**Findings:**

The attractiveness of alternatives significantly enhance medical personnel’s shift intention to rural areas, and the factor of personal innovation also has a positive effect on the shift intention of medical personnel, while the effect of subjective norms was not significant, and results of social factors show a negative correlation rather than the expected positive one. In contrast, dissatisfaction and shift barriers exerted pronounced negative effects. These findings provide theoretical grounding for promoting downward shift intention among healthcare talent and lay the foundation for advancing healthy lifestyles and preventive healthcare concepts in rural regions.

**Conclusion:**

This study precisely identifies the key factors influencing medical personnel’s intentions to shift, providing empirical support for optimizing rural healthcare talent retention systems, enhancing rural medical quality, and narrowing the gap in healthcare resources between urban and rural areas. It also lays a theoretical foundation for relevant policy formulation, rural health education, and improving residents’ health literacy.

## 1 Introduction

In recent years, the development of rural healthcare in China has faced many challenges, including a lack of human resources, an imbalance in the allocation of urban and rural healthcare resources, insufficient equipment, and an unsound rural healthcare talent protection system (1). The 2020 Statistical Bulletin of China’s Hygiene and Health Development shows that by the end of 2020, rural doctors had reached 747,000 and 509,000 administrative villages. On average, there are less than 2 rural doctors per village. Statistics for the past five years show that the number of rural doctors has plummeted at an average rate of nearly 50,000 per year. According to the 2022 China Statistical Yearbook of Hygiene and Health, 28.8% of village doctors are 60 years old or older, and 74.9% are 45 years old or older. And data released in the 2023 China Statistical Yearbook shows that in 2022, there were more than 660,000 village doctors and health workers in China, while the rural population exceeded 500 million, and village doctors were generally older and less educated, further highlighting the dire situation of human resources in the field of rural healthcare (2). In order to cope with the shortage of professionals in the rural medical field, the No.1 Document of the Central Government in 2024 explicitly pointed out the need to promote the comprehensive revitalisation of the countryside, whose core strategies include: strengthening the construction of the rural talent team, implementing the special plan for revitalising the countryside with talents, increasing the cultivation of local talents in the countryside, guiding the flow of professional and technical talents from urban areas to the countryside in an orderly manner, and comprehensively upgrading the comprehensive quality of the farmers.

In this context, this paper uses the push-pull-mooring model as a tool to explore the following questions: in the absence of professional talents in the rural medical field, how to explore effective mechanisms to promote the sinking of medical personnel to the countryside, narrow the gap between urban and rural medical levels, and improve the overall quality of public health services? In addition, we hope the conclusions drawn from this study will provide solid empirical support for the formulation and improvement of relevant policies, so as to promote the efficient shift intention and rational allocation of medical personnel, establish a rural medical personnel protection system, and promote the balanced development of urban and rural medical resources.

## 2 Literature review and research hypothesis

### 2.1 Theoretical basis and application of push-pull anchoring model (PPM)

The push-pull-mooring model (PPM) is a classic theoretical framework for the study of population migration, originally proposed by American scholar E. S. Lee (3) in the 1960s. Lee categorised the factors affecting population migration into two aspects: ‘push’ and ‘pull’. “Push” refers to factors that motivate individuals to leave their original place of residence, such as poor working conditions and limited career development. The ‘pull’ refers to the factors that attract individuals to move to a new place of residence, such as better job opportunities and higher income, etc. Moon (4) further extends the model by introducing anchoring factors, which are considered to potentially facilitate or impede population migration, resulting in a more comprehensive push-pull anchoring model. The model is not only applicable to the study of population migration, but is also widely used in the analysis of migration behaviours in other fields, such as the migration behaviours of social media users (5). In the field of social media, scholars draw on the PPM model to study the migration behaviour of users between different platforms. For example, Cao (6) earlier paid attention to the phenomenon of user migration between different products under the same medium and studied the migration behaviour of users from blogs to microblogs. Zhou et al. (7) further explored the migration behaviour of social media users based on the PPM model. Deng et al. (8) found that in mobile short video platforms, relationship conversion cost, procedural cost, and usage habits are important barriers affecting users’ intention to migrate. Among them, the increase of relationship conversion cost due to the lack of relationship network greatly hinders users’ shift intention.

### 2.2 Current situation and challenges of urban–rural medical personnel shift intention

The issue of urban–rural medical personnel shift intention has always been the focus of academic attention. The shortage of medical personnel in rural areas of China is a serious problem and is characterised by aging and low education (9). Through various theoretical frameworks and policy studies, scholars have attempted to reveal the intrinsic mechanism of medical personnel shift intention and provide a basis for policy formulation. For example, Yang et al. (10) analysed through questionnaires and conditional Logit models, they found that factors such as income, domestic first-class platforms, and the environment of the talent system have a significant impact on the career preference of high-level health talents. In addition, Yuan et al. (11) studied the application mechanism of O2O model in the flow of urban and rural medical talents under the background of rural revitalisation, and proposed to learn from the international experience of online medical consultation, to promote the Internet medical services to benefit the farmers, to improve the online and offline service chain of rural medical care, and to build a complete medical service system and cooperation and coordination mechanism. Chen et al. (12) analysed the internal and external factors affecting medical students’ intention to work in rural areas by means of a questionnaire survey. The results of the study showed that gender, whether they are oriented medical students, family economic status, whether they have rural internship experience, salary and social security, working and living conditions, development space and employment prospects are the key factors influencing medical students’ intention to work in rural grassroots. In recent years, with the promotion of the rural revitalisation strategy, some scholars have begun to pay attention to the cultivation and introduction mechanism of rural medical personnel, and have put forward suggestions such as strengthening the cultivation of rural medical personnel and optimising the rural medical working environment. However, most of the existing studies focus on the description of the current situation and problem analysis, lacking in-depth theoretical exploration and empirical research.

### 2.3 Research hypothesis and theoretical model

**Figure 1:**
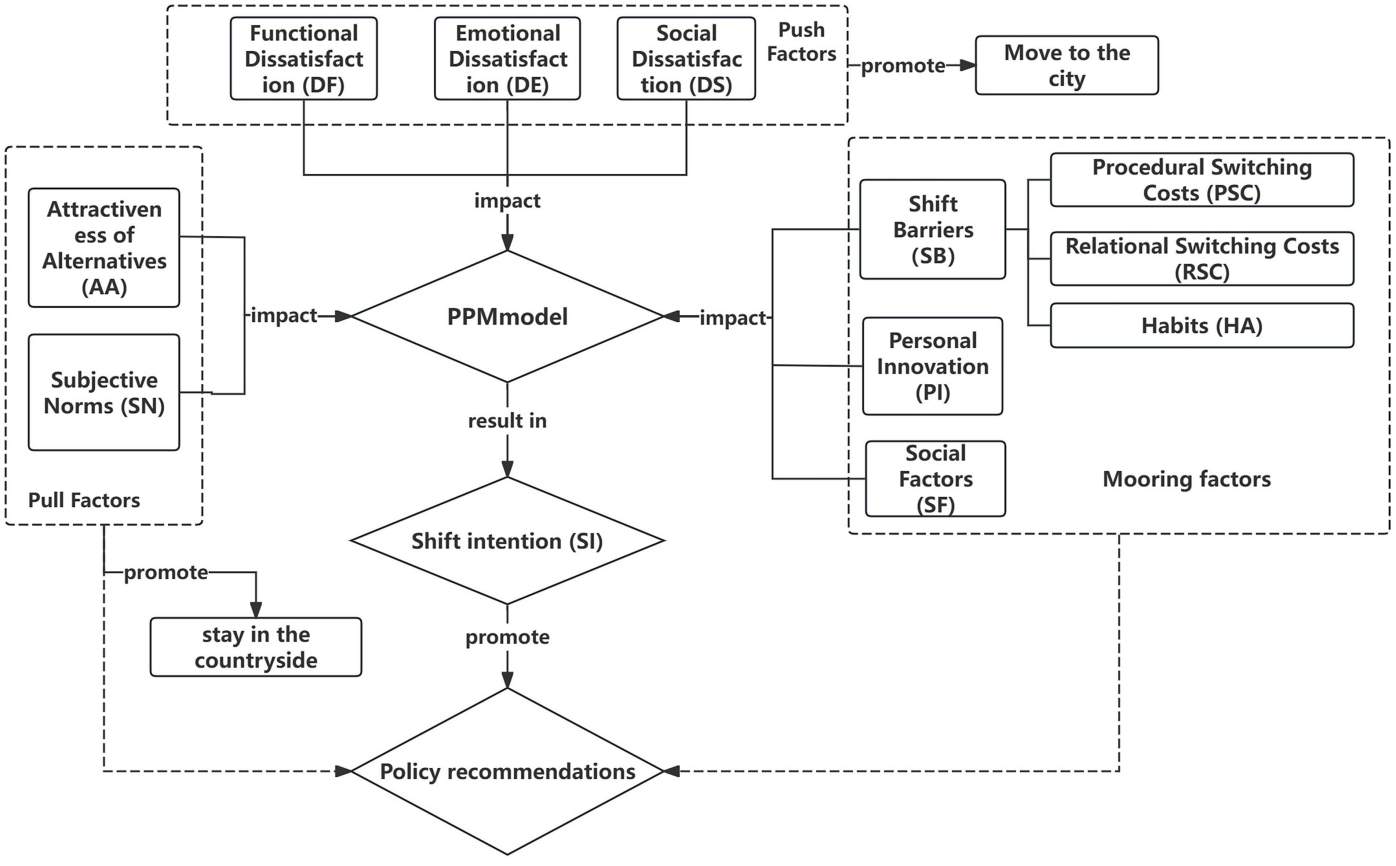
Theoretical framework of the study.

The study constructs a theoretical analysis framework on the basis of the push-pull-mooring Model (as shown in Figure 1), and synthesizes the various influencing factors mentioned in the existing studies to analyse and explore the differences in the shift intention of different groups, with the aim of providing the academic research in the field of urban–rural medical personnel shift intention with a new theoretical perspective.

#### 2.3.1 Driving factor—dissatisfaction degree

The degree of satisfaction of medical personnel with the transfer work is an important factor in determining their intention to shift to rural areas. Specifically, the lower the satisfaction with the rural work environment, the weaker the shift intention and thus the more inclined to give up the decision to shift.

On the basis of existing studies, this paper will start from the three dimensions of dissatisfaction, i.e., functional satisfaction, emotional satisfaction and social satisfaction, to explore their effects on medical personnel’s transfer behavioural intention. The first is functional satisfaction, i.e., medical personnel’s satisfaction with rural infrastructure as well as medical equipment dimensions. As Li′s study points out, one of the main challenges facing the revitalisation of rural talents is the lack of infrastructure, which will directly affect the attractiveness of the countryside to talents (13). Secondly, emotional satisfaction refers to the degree of medical personnel’s satisfaction with the emotional experience of work and life. Huang and Chen’s study shows that job satisfaction, organisational commitment and burnout have a significant impact on primary healthcare workers’ intention to leave their jobs, and these factors will further affect medical personnel’s shift intention (14). Lastly, social satisfaction, which is the degree of need fulfillment of medical personnel in social and interpersonal interactions, Liu pointed out in his study that tension between doctors and patients and disharmony in colleague relations are one of the important reasons leading to the shortage of primary healthcare talents, which will directly affect the turnover rate of medical talents (15). Based on this, this paper proposes the following hypotheses:

*H1:* Dissatisfaction with the overall environment of rural healthcare will have a negative impact on the shift intention of medical personnel.

#### 2.3.2 Tension factor-attractiveness

In addition to the dissatisfaction of medical personnel with the overall environment of rural healthcare, factors such as the support of relevant policies and the relatively weak competitiveness of rural healthcare positions will also lead to the intention of medical personnel to transfer. Research by Li (16) and others has already shown that, in the context of market competition, the introduction of public investment and major projects will trigger the shift intention of talents and create an employment transfer effect. In addition, perceptions of social groups (e.g., family, lovers, friends, etc.) can also have an impact on medical personnel’s shift intention employment due to factors such as family responsibilities and kinship. Therefore, this study examines the attractiveness of alternatives and subjective norms as part of pull factors.

#### 2.3.3 Attractiveness of alternatives

‘Attractiveness of alternatives’ refers to the unique advantages and attractiveness of rural medical positions over other employment options such as urban hospitals. Medical personnel consider working in rural areas because rural medical positions can meet their career development and livelihood needs. When medical personnel realise that the government’s financial and policy support for rural healthcare is better, they are more inclined to choose to work in rural medical positions. Under the current background of actively promoting the strategy of rural revitalisation, the development of the rural medical field has been supported and incentivised by a number of policies, and rural medical workers are able to enjoy more financial subsidies and welfare benefits. Under the current fierce social competition for jobs, the job market provides relatively limited investment and employment opportunities, and there is a certain degree of exclusivity between various types of investments and projects. These advantages and attractions of rural healthcare jobs are therefore of particular importance. Given that public investments and major projects are usually led by the government, the rural healthcare sector has a unique advantage in terms of policy support and resource allocation. This advantage makes rural medical jobs more competitive in meeting the employment and livelihood needs of medical professionals and improving the quality of medical services. Accordingly, this study regards the attractiveness of alternatives as a key pull factor, and on this basis puts forward the hypothesis that more perfect support policies and subsidies can significantly stimulate the intention of medical personnel to shift to rural medical positions. Based on the above analysis, this paper proposes the following hypothesis:

*H2:* The attractiveness of alternatives has a positive effect on the shift intention of medical personnel.

### 2.4 Subjective norms

Subjective norms refer to the social expectations and pressures felt by individuals when deciding whether to adopt a certain behaviour. In the current increasingly competitive job market, the employment pressure in the healthcare industry is particularly acute. With the continuous growth of the population to be employed and the unemployed, medical professionals are facing unprecedented career challenges. In addition, the development of rural healthcare is not only a local issue, but also an inevitable requirement for the progress of the country, and the link between individual employment and national development has become increasingly close. When family, friends, colleagues and social networks actively advocate for medical personnel to devote themselves to rural healthcare, and the government creates favourable conditions for medical personnel by optimising the rural working environment and providing policy support, these factors will together stimulate medical personnel’ s interest in rural healthcare work and lead to a positive shift in their professional behaviour. Therefore, this study concludes that subjective norms are the key motivators that drive medical personnel to rural areas and proposes the following hypothesis:

*H3:* Subjective norms have a positive effect on medical personnel’s shift intention to rural areas.

#### 2.4.1 Anchoring factors

Given the complexity of transfer behaviours, a single push-pull theory cannot fully explain individual shift intentions and decision-making processes. Factors such as individual behaviour, cultural background or social environment may also facilitate or hinder shift intention and decision-making, which corresponds to the anchoring factors in the PPM model, e.g., elements such as shift barriers and personal innovation may play a role in an individual’s shift intention. Adaptation to urban life and the cost of living to be borne in the countryside may weaken individuals’ motivation to move. In addition, personal attributes such as the increasing quality of urban healthcare services, the widening gap between urban and rural healthcare services, personal innovation preferences and past experiences may also play a role in fixing transfer behaviour. Therefore, this study included shift barriers, personal innovation and social factors in the examination of anchoring factors.

##### 2.4.1.1 Shift barriers

In this study, conversion barriers were defined as the economic, social, and psychological costs that healthcare workers have to bear when changing to the status of a township physician (17). According to the authors, shift barriers can be measured and gauged in terms of dimensions such as job costs, interpersonal costs, personality and perceived risk. Taking into account the differences and characteristics of urban and rural healthcare, this paper focuses on the dimensions of switching costs and habits. Transfer cost refers to healthcare workers’ subjective assessment of the costs they need to bear to change their work environment. Previous studies usually regard transfer costs as an anchor factor that inhibits transfer behaviour, thus reducing the likelihood of healthcare professionals moving to township areas. For example, the lower cost of living in townships, although attractive, may not be sufficient to compensate for the potential loss of income when medical professionals move to townships; the limitations of healthcare organisations in townships in terms of providing opportunities for professional development and continuing education may limit the career prospects of medical professionals; and the fact that medical professionals have built up a wide range of social networks in the city, including colleagues, friends, and family members, and leaving these networks may lead to feelings of isolation and social distance, etc. Burnham classifies switching costs into three categories: procedural switching costs, financial switching costs and relationship switching costs (18). Procedural switching costs can be understood as the psychological adjustment stress and discomfort experienced by an individual in the process of adapting to a new environment. Financial costs refer to the economic losses and loss of experience gained as a result of moving to a rural area for employment. Relationship costs refer to the disruption of existing social networks and the cost of establishing new relationships as a result of the change in workplace. When healthcare workers perceive the costs to be too high, their shift intention decreases.

##### 2.4.1.2 Habituation

It is commonly defined in psychology as an automated, unconscious pattern of repetitive behaviour. When healthcare professionals become accustomed to their current work environment, work patterns, and community culture, they tend to be less motivated to seek other job opportunities or relocate to a new environment. This habituation includes not only familiarity with work processes, but also social relationships and the pace of life. Thus, habituation can be seen as a psychological anchoring factor that limits healthcare professionals’ intention to transfer to some extent. Based on this, we propose the following hypothesis:

*H4:* Shift barriers have a negative impact on healthcare professionals’ shift intentions.

*H4a:* Procedure-based costs have a positive effect on the formation of shift barriers.

*H4b:* Relationship-based costs have a positive effect on the formation of conversion barriers.

*H4c:* healthcare professionals’ habituation to the current work environment has a positive effect on the formation of shift barriers.

##### 2.4.1.3 Personal innovation

In this paper, personal innovation is defined as medical personnel’s acceptance of transferring from urban hospitals and research institutes to work in township hospitals and their intention to devote themselves to work in rural healthcare services. Some studies have pointed out that people with higher innovative ability are more willing to try new work environments and challenges, and thus they are relatively more likely to transfer to new work environments (e.g., working in townships). This innovativeness is reflected not only in the acceptance of new technologies and methods, but also in the ability to adapt and openness to new work environments. Based on this, the following hypothesis is proposed:

*H5:* Personal innovation has a positive effect on medical personnel’s shift intention to rural areas.

##### 2.4.1.4 Social factors

The social factors in this paper refer specifically to the preference of medical personnel for the stability of the work environment when choosing a career. In the post-epidemic era, people’s emphasis on career stability has increased significantly, while rural areas provide a more stable career environment and development space due to relatively low competitive pressure and strong policy support. Such an environment is attractive to healthcare workers who seek stability. Therefore, it can be hypothesised that those healthcare professionals who highly value career stability may be more inclined to choose rural areas for employment. Based on this, the following hypothesis is proposed:

*H6:* Social factors have a positive influence on medical personnel’s intention to go to rural areas.

## 3 Research design

### 3.1 Questionnaire design and variable measurement

This study selects five representative districts in Zhejiang Province as the research area, takes medical professionals and medical students as the research objects, adopts the questionnaire survey method to verify the constructed research model. Based on the PPM model, the study first systematically combed the domestic and international literature on transfer behaviours, and preliminarily screened out the question items related to the intention of medical personnel to transfer. In order to verify the validity of the questions and explore the potential key influencing factors, the study conducted in-depth interviews with 22 healthcare practitioners from different fields, including 2 pharmacists, 5 doctors and 15 medical students. Based on the results of the interviews and taking into account their perceptions of the factors influencing medical brain drain, specific questions in the questionnaire were tailored (see Table 1).

**Table 1:** Measurement items and sources of variables in the model.

Variables		Survey	Source
Pushing factors	Functional dissatisfaction (DF)	I think the medical equipment in the townships is very poorly equipped and has a lot of imperfections	Li et al. (22)
		The current infrastructure in townships is inadequate	
		It is difficult to access resources and opportunities for in-depth learning in the township work environment.	
		The current township medical equipment does not allow me to do my job well	
		Current township medical equipment does not allow me to do my job well	
	Emotional dissatisfaction (DE)	Working in the Township lacks appeal to me.	Li (23)
		Daily life in the countryside is boring and it is difficult for me to find happiness.	
		I think the policy of supporting medical staff to go to the countryside does not meet my needs.	
		Working in the countryside cannot reduce my study/work pressure	
	Social dissatisfaction (DS)	It is difficult to realise my self-worth working in a rural hospital	Li and Frankel (29)
		I think it is difficult to have a rich social life when working in a township hospital.	
Pulling factors	Attractiveness of alternative choices (AA)	If I had a choice, I would prefer to work in a township hospital or other organisations	Kim et al. (17, 24)
		I think working in a rural area would make life simpler and easier.	
		Working in rural hospitals and other institutions would give me more satisfaction in my work, life and comfort.	
		Working in township hospitals and other institutions will give me advantages that are not available in urban jobs.	
		Employment opportunities in township hospitals give me a more pronounced sense of self-fulfilment	
		Under the current policy framework, township hospitals have an advantage over urban hospitals and research institutes in meeting individual needs for self-actualisation and providing self-recognition.	
	Subjective norms (SN)	In the current employment environment, people who are important to me (e.g., relatives, lovers, friends) want me to choose to work in an organisation such as a township hospital.	Frankel et al.
		In the current employment environment, people who are important to me (e.g., relatives, lovers, friends) suggest that I choose to be employed in an institution such as a rural hospital	
Mooring factors	Shift barriers (SB)	When I go to work in a rural area, I will lose the relationships I have in my current area	Kim et al. (17, 24)
		Perceived higher risk when I move to a rural area for work	
	Procedure Switching Costs (PSC)	Familiarisation with rural healthcare takes a process.	Zhang and Liu (25)
		Moving to a rural area takes time to become familiar with the people and environment of the township	
	Relationship Switching Cost (RSC)	Moving to a rural job can result in the loss of current close colleagues.	Burnham et al. (18)
		Moving to a rural job would lose my current role models	
		Moving to a rural area will require me to spend some time and effort meeting new people	
	Habituation (HA)	I am used to working in my current environment.	Ye (26)
		Working in my current environment comes naturally to me.	
		I feel that I depend on my current work environment	
	Personal Innovation (PI)	I like to try new environments and jobs.	Agarwal and Prasad (27)
		Among my friends, I am the one who likes to try new jobs.	
		I usually do not have much hesitation in trying new things and jobs.	
	Shift Intentions (SI)	I am taking the initiative to learn about policies and mechanisms related to medical work in the townships	Kim et al. (17, 24)
		I have started to go to township hospitals for a change.	
		It is very likely that I will work in a township hospital	
		I have decided to work in a township hospital	
	Social factors (SF)	After the epidemic, I think high job stability is more attractive than high salary	Wang et al. (28)
		After the epidemic, I will choose a job with high stability.	

After finalisation, the medical personnel Shift Intention Influencing Factors Scale had 38 items, and a five-point Likert scale was used to quantitatively rate all survey items.

### 3.2 Data collection

This study was conducted by “online + offline” survey, a total of 430 questionnaires were distributed, and finally 413 valid questionnaires were recovered, with a recovery rate of about 96%, of which 210 were for medical professionals and 203 were for medical students, as shown in the statistical description of the sample in Table 2. There were 98 males, accounting for 23.7%, and 315 females, accounting for 76.3%. The age and occupational status of the sample showed that medical professionals were proportionately more balanced in age distribution and occupational status distribution, and medical students were mostly under 25 years old. In terms of educational attainment, 87.7% had a bachelor’s degree or higher, so representation in the wider population may be more limited.

**Table 2:** Descriptive statistical analysis of the sample (*N* = 413).

Item	Classification	Number		Proportion	
		Medical professionals (N1 = 210)	Medical students (N2 = 203)	Medical professionals (50.8%)	Medical students (49.2%)
Sex	Male	64	34	30.5%	16.7%
	Female	146	169	69.5%	83.3%
Age: 25 years and below	25 years and below	50	177	23.8%	87.2%
	26–35 years	93	17	44.3%	8.4%
	36–45 years	39	0	18.6%	0%
	45 years and over	28	9	13.3%	4.4%
Occupational status	Working in a township medical institution	122	/	58.1%	/
	Working in urban medical institutions	88	/	41.9%	/
Educational level	College and below	33	18	15.7%	8.9%
	Undergraduate	109	142	51.9%	70.0%
	Master’s Degree	61	38	29.1%	18.7%
	PhD	7	5	3.3%	2.5%

### 3.3 Reliability test

#### 3.3.1 Descriptive statistical analysis of each variable

Table 3 shows that all 12 core variable scores are within the Likert-5 point scale range. The mean values of “Attractiveness of Alternative Choices” (AA) and “Subjective Norms” (SN) were above the midpoint, indicating that the sample perceived positively external choices and significant others’ support; the mean values of the variables of the three categories of dissatisfaction and switching costs were low, indicating that dissatisfaction and resistance were at a moderately low level, but with sufficient individual differences. The skewness and kurtosis of the data are well controlled, meeting the requirements of normal distribution, effectively reducing the parameter estimation bias, and guaranteeing the reasonableness of the subsequent analyses.

**Table 3:** Basic indicators.

Name	Sample size	Minimum value	Maximum value	Mean ± standard deviation	Kurtosis	Skewness
DF	413	1	5	2.739 ± 1.085	−1.128	0.358
DE	413	1	5	2.611 ± 0.997	−0.872	0.433
DS	413	1	4.75	2.481 ± 0.926	−0.284	0.651
AA	413	1	5	3.324 ± 1.089	−1.059	−0.463
SN	413	1	5	3.416 ± 1.082	−0.675	−0.597
SB	413	1	5	2.597 ± 1.025	−0.628	0.524
PSC	413	1	5	2.733 ± 1.102	−0.924	0.444
RSC	413	1	5	2.623 ± 0.998	−0.806	0.453
HA	413	1	5	2.622 ± 1.061	−0.742	0.488
PI	413	1	5	3.398 ± 0.975	−0.600	−0.591
SI	413	1	5	3.462 ± 0.956	−0.667	−0.527
SF	413	1	5	2.490 ± 0.979	−0.462	0.61

#### 3.3.2 Reliability analysis

Table 4 Cronbach’s alpha coefficient was used to assess the internal consistency of the questionnaire, and the results showed that the alpha coefficients of the 12 core variables were higher than 0.7 (range 0.823–0.892), which meets the acceptable standard of reliability for social science research. Among them, the alpha coefficient of “attractiveness of alternative choices” (AA) reaches 0.892, indicating a high degree of consistency among the six items; the lowest one, “social factors” (SF), is also 0.823, which verifies the overall stability of the research instrument. This table provides a reliable reliability support for the subsequent structural equation modelling analysis.

**Table 4:** Cronbach’s reliability analysis.

Variables	Number of items	Cronbach’s alpha coefficient
Functional dissatisfaction (DF)	5	0.886
Emotional dissatisfaction (DE)	4	0.876
Social dissatisfaction (DS)	4	0.846
Attractiveness of alternatives (AA)	6	0.892
Subjective norms (SN)	4	0.871
Shift Barriers (SB)	3	0.850
Programme Switching Costs (PSC)	3	0.873
Relationship Conversion Cost (RSC)	4	0.858
Habits (HA)	3	0.866
Personal Innovation (PI)	4	0.871
Shift Intention (SI)	4	0.858
Social factors (SF)	3	0.823

#### 3.3.3 Validated factor analysis of CFA

In this paper, AMOS 24.0 was used to conduct validation factor analysis, and the results are as follows. From Table 5, it can be seen that: the CMIN/DF of the medical student model is 1.373, which is less than 3 and reaches the good standard; the IFI, TLI, and CFI are 0.929, 0.919, and 0.928, which are greater than 0.9 and reach the good standard; the RMSEA is 0.043, and the RMR is 0.067, which is less than 0.08, and reach the good standard; it shows that the model has good fitness and can be further analyzed.

**Table 5:** Medical student model fitting indicators.

Indicators	CMIN/DF	RMR	IFI	TLI	CFI	RMSEA
Common indicators	<3	<0.08	>0.9	>0.9	>0.9	<0.08
Measurement model fit results	1.373	0.067	0.929	0.919	0.928	0.043

From Table 6, it can be seen that: the CMIN/DF of the medical professionals model is 1.418, which is less than 3 and reaches the good standard; the IFI, TLI, and CFI are 0.936, 0.927, and 0.935 respectively, which are greater than 0.9 and reach the good standard; the RMSEA is 0.045, and the RMR is 0.071, which is less than 0.08 and reaches the good standard; it shows that the model has good fitness and can be further analysis.

**Table 6:** Medical professionals model fit indicators.

Indicator	CMIN/DF	RMR	IFI	TLI	TLI	RMSEA
Common indicators	<3	<0.08	>0.9	>0.9	>0.9	<0.08
Measurement model fitting results	1.418	0.071	0.936	0.927	0.935	0.045

Table 7 shows Composite reliability and the convergent validity. Composite reliability (CR) is one of the criteria for assessing the internal quality of the model, reflecting whether all items under each latent variable consistently explain that latent construct. IFCR≥0.70, generally considered to indicate good reliability (19, 20), IF 0.60 ≤ CR < 0.70, it can be acceptable in exploratory or early-stage research; if CR < 0.60, it means indicates insufficient internal consistency of the latent construct, suggesting that items with low factor loadings should be removed or the model should be re-specified. As shown in Table 7, all CR values exceed 0.7, indicating that the items within each latent variable consistently account for the corresponding construct. The convergent validity of each dimension is reflected by the average variance extracted (AVE), which is commonly used to evaluate the convergent validity of a scale. AVE directly shows the proportion of variance in the observed variables explained by the latent construct relative to measurement error. A higher AVE value indicates that a greater proportion of variance is explained by the latent construct, while measurement error is relatively lower. Conventionally, a threshold of 0.5 is considered acceptable. As shown in Table 7, all AVE values exceed 0.5. These results demonstrate that the questionnaire’s structural model possesses good convergent validity.

**Table 7:** Convergent validity of the model.

Latent variable		Measurement items	Criterion loadings		AVE&CR		Cronbach’s α	
			Medical professionals	Medical students	Medical professionals	Medical students	Medical professionals	Medical students
Pushing factors	Functional dissatisfaction (DF)	DF1	0.846	0.751	0.664 0.908	0.549 0.859	0.908	0.858
		DF2	0.789	0.736				
		DF3	0.818	0.689				
		DF4	0.811	0.777				
		DF5	0.808	0.748				
	Emotional dissatisfaction (DE)	DE1	0.824	0.785	0.695 0.901	0.566 0.839	0.901	0.837
		DE2	0.864	0.684				
		DE3	0.810	0.731				
		DE4	0.835	0.804				
	Social dissatisfaction (DS)	DS1	0.793	0.764	0.627 0.870	0.533 0.820	0.869	0.818
		DS2	0.760	0.693				
		DS3	0.771	0.698				
		DS4	0.841	0.762				
Pulling factors	Attractiveness of alternatives (AA)	AA1	0.766	0.707	0.634 0.912	0.521 0.867	0.912	0.866
		AA2	0.781	0.680				
		AA3	0.783	0.680				
		AA4	0.809	0.747				
		AA5	0.821	0.731				
		AA6	0.815	0.781				
	Subjective norms (SN)	SN1	0.784	0.774	0.638 0.876	0.620 0.867	0.875	0.866
		SN2	0.822	0.791				
		SN3	0.803	0.768				
		SN4	0.785	0.815				
Anchoring factors	Shift barriers (SB)	SB1	0.815	0.805	0.649 0.847	0.644 0.844	0.846	0.844
		SB2	0.785	0.790				
		SB3	0.816	0.812				
	Programme conversion cost (PSC)	PSC1	0.787	0.796	0.731 0.890	0.664 0.855	0.889	0.855
		PSC2	0.868	0.848				
		PSC3	0.905	0.799				
	Relationship switching costs (RSC)	RSC1	0.799	0.838	0.626 0.870	0.575 0.843	0.869	0.843
		RSC2	0.827	0.760				
		RSC3	0.750	0.625				
		RSC4	0.788	0.794				
	Habituation (HA)	HA1	0.830	0.843	0.683 0.866	0.684 0.867	0.865	0.866
		HA2	0.873	0.800				
		HA3	0.774	0.838				
	Personal Innovation (PI)	PI1	0.835	0.730	0.652 0.882	0.604 0.859	0.881	0.858
		PI2	0.797	0.786				
		PI3	0.835	0.766				
		PI4	0.760	0.825				
	Shift Intention (SI)	SI1	0.841	0.756	0.662 0.887	0.512 0.807	0.887	0.809
		SI2	0.837	0.737				
		SI3	0.775	0.672				
		SI4	0.800	0.693				
	Social factors (SF)	SF1	0.741	0.689	0.633 0.838	0.595 0.814	0.834	0.809
		SF2	0.838	0.794				
		SF3	0.805	0.823				

Specifically, the test results for the medical student sample showed that the combined reliability (CR) of all the latent variables was significantly higher than 0.7 (range 0.807–0.867), with a CR of 0.807 for “Shift Intention” (SI) and 0.867 for “Attractiveness of Alternatives “(AA), which indicated that the items explained the latent variables more consistently, and that the average variance extraction (AVE) was above 0.5 (range 0.512–0.684), with “Shift Intention” (SI) and “Habituation” (HA) being the most important ones. The average variance extracted (AVE) exceeded 0.5 (range 0.512–0.684), with AVE = 0.512 as the minimum for “Shift Intention” (SI) and AVE = 0.684 as the maximum for “Habituation” (HA), indicating good control of measurement error; for example, the standardised factor loadings for “functional dissatisfaction” (DF) were above 0.6 (range 0.689–0.777, *p* < 0.001), further validating the high degree of convergence of the measurement items.

The test results for the sample of medical professionals showed that the combined reliability (CR) of all latent variables was significantly higher than 0.8 (range 0.838–0.912), with CR values of 0.838 for “Social factors” (SF) and 0.912 for “Attractiveness of Alternatives” (AA), which indicated that the items had a strong consistency in explaining the latent variables; the average variance extraction (AVE) was higher than 0.6 (range 0.626–0.731), with AVE = 0.626 as the minimum value for “Relationship switching costs “(RSC) and AVE = 0.731 as the maximum value for “Programme Conversion Cost” (PSC), which indicates that the control of measurement error is excellent; for example, the standardised factor loadings of “functional dissatisfaction” (DF) were all higher than 0.7 (range 0.789–0.846, *p* < 0.001), and the aggregation of measurement items is significantly better than that of the medical student group, providing a robust measurement basis for cross-group comparison.

One method of testing discriminant validity is to compare the correlation coefficients with the square root of the average variance extracted (AVE). The rationale is that each construct has an AVE value as well as correlation coefficients with other constructs. If the square root of a construct’s AVE is greater than its correlations with other constructs, discriminant validity is considered satisfactory.

Tables 8, 9 show the discriminant validity of medical students and medical professionals. In Table 8, the square roots of the average variance extracted (AVE) for each latent variable (range 0.715–0.827) were significantly higher than the absolute values of their correlation coefficients with the other latent variables (range −0.702–0.553), indicating that the measurement model discriminated well between the different constructs within the medical student population. For example, the square root of the AVE for “functional dissatisfaction” (DF) was 0.741, which was higher than the correlation coefficient of 0.722 with “Attractiveness of alternatives” (AA), indicating that medical students were able to clearly differentiate between functional deficits and affective responses on career attitudes.

**Table 8:** Distinguishing validity (medical students).

	DF	DE	DS	AA	SN	SB	PSC	RSC	HA	PI	SI	SF
DF	**0.741**											
DE	0.372	**0.752**										
DS	0.364	0.51	**0.73**									
AA	−0.414	−0.365	−0.476	**0.722**								
SN	−0.446	−0.503	−0.456	0.473	**0.787**							
SB	0.301	0.278	0.264	−0.386	−0.324	**0.802**						
PSC	0.426	0.42	0.555	−0.504	−0.513	0.308	**0.814**					
RSC	0.326	0.421	0.366	−0.355	−0.3	0.238	0.402	**0.758**				
HA	0.375	0.367	0.411	−0.371	−0.345	0.153	0.345	0.404	**0.827**			
PI	−0.301	−0.302	−0.446	0.386	0.319	−0.176	−0.406	−0.311	−0.33	**0.777**		
SI	−0.547	−0.579	−0.702	0.617	0.553	−0.378	−0.596	−0.502	−0.427	0.542	**0.715**	
SF	0.394	0.328	0.346	−0.384	−0.442	0.287	0.313	0.284	0.271	−0.235	−0.384	**0.771**

Bolded numbers on the diagonal are AVE square root values.

**Table 9:** Distinguished validity (medical professionals).

	DF	DE	DS	AA	SN	SB	PSC	RSC	HA	PI	SI	SF
DF	**0.815**											
DE	0.228	**0.834**										
DS	0.254	0.192	**0.792**									
AA	−0.4	−0.103	−0.231	**0.796**								
SN	−0.511	−0.185	−0.332	0.366	**0.799**							
SB	0.518	0.25	0.362	−0.405	−0.497	**0.806**						
PSC	0.384	0.247	0.339	−0.219	−0.394	0.429	**0.855**					
RSC	0.398	0.189	0.325	−0.347	−0.532	0.416	0.349	**0.792**				
HA	0.327	0.156	0.352	−0.311	−0.337	0.365	0.242	0.317	**0.827**			
PI	−0.108	0.039	0.046	−0.049	0.188	−0.052	−0.089	−0.057	−0.056	**0.807**		
SI	−0.437	−0.203	−0.234	0.307	0.36	−0.48	−0.219	−0.332	−0.303	0.02	**0.814**	
SF	0.4	0.144	0.427	−0.392	−0.505	0.44	0.371	0.539	0.39	−0.115	−0.495	**0.796**

Bolded numbers on the diagonal are AVE square root values.

In Table 9, the AVE square root of each latent variable (range 0.792–0.855) significantly exceeded the absolute value of its correlation coefficients with the other latent variables (range −0.532–0.539), suggesting that occupational experience strengthens the measurement model’s ability to discriminate between complex constructs. For example, the square root of the AVE for ‘functional dissatisfaction’ (DF) was 0.815, which was higher than its correlation coefficient with ‘Attractiveness of alternatives’ (AA) of 0.796, reflecting a more refined assessment of the differentiation between functional deficits and emotional responses.

In the present analysis, the square roots of the AVE values for all constructs exceeded the absolute values of the inter-construct correlations, indicating good discriminant validity.

### 3.4 Correlation analysis

Analysing the relationship between shift intention (SI) and the other 11 variables by Pearson’s correlation coefficient (Table 10), we found that SI was significantly negatively correlated with functional dissatisfaction (DF), emotional dissatisfaction (DE), and social dissatisfaction (DS) (r = −0.485 to −0.429, *p* < 0.01), suggesting that service deficiencies inhibit the intention to shift; and was significantly positively correlated with the attractiveness of the alternative choice (AA) and subjective norms (SN) were significantly positively correlated (r = 0.435 ~ 0.443, *p* < 0.01), suggesting that external opportunities and social acceptance promote transfer behaviour; meanwhile, SI was significantly negatively correlated with shift barriers (SB), procedural switching costs (PSC), relational switching costs (RSC), and habits (HA) (r = −0.442 ~ −0.354, *p* < 0.01), reflecting the hindering effect of cost and inertia on transfer; while the positive correlation between personal innovation (PI) and SI (r = 0.242, *p* < 0.01) and the negative correlation between social factors (SF) and SI (r = −0.452, *p* < 0.01) further reveal the differential effects of psychological traits and environmental pressures on transfer decisions.

**Table 10:** Pearson correlation.

Pearson correlation												
	DF	DE	DS	AA	SN	SB	PSC	RSC	HA	PI	SI	SF
DF	1											
DE	0.283**	1										
DS	0.304**	0.321**	1									
AA	−0.407**	−0.209**	−0.339**	1								
SN	−0.483**	−0.315**	−0.389**	0.414**	1							
SB	0.423**	0.249**	0.319**	−0.393**	−0.418**	1						
PSC	0.403**	0.318**	0.437**	−0.348**	−0.449**	0.369**	1					
RSC	0.370**	0.280**	0.346**	−0.351**	−0.428**	0.346**	0.373**	1				
HA	0.349**	0.242**	0.379**	−0.338**	−0.341**	0.265**	0.289**	0.356**	1			
PI	−0.197**	−0.101*	−0.176**	0.146**	0.250**	−0.118*	−0.234**	−0.177**	−0.179**	1		
SI	−0.485**	−0.343**	−0.429**	0.435**	0.443**	−0.442**	−0.378**	−0.408**	−0.354**	0.242**	1	
SF	0.401**	0.216**	0.392**	−0.389**	−0.478**	0.379**	0.345**	0.429**	0.336**	−0.174**	−0.452**	1

* *p* < 0.05; ** *p* < 0.01.

### 3.5 Constructing structural equation models

**Figure 2:**
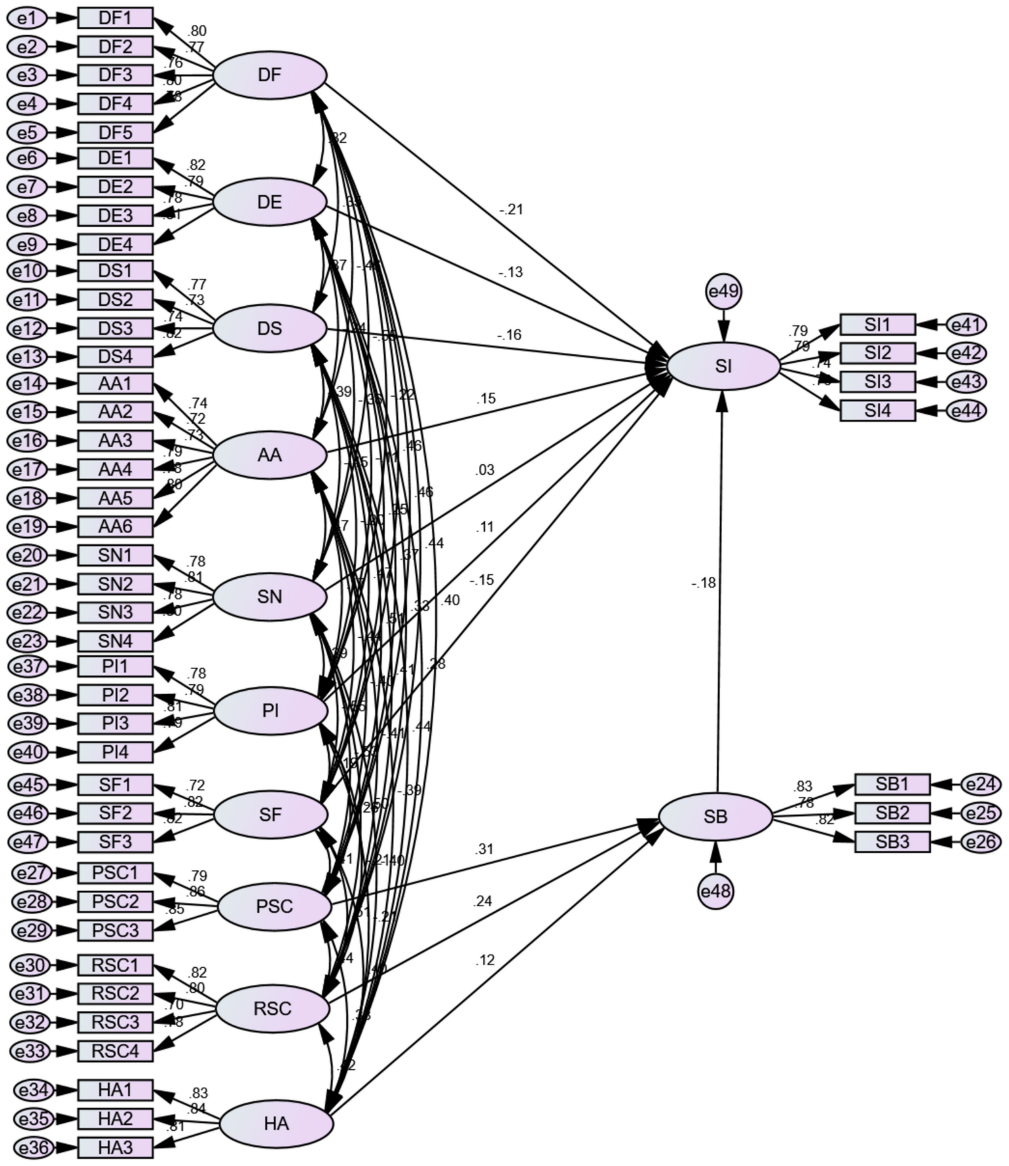
Model test results.

In this study, we used Amos24.0 software to conduct an in-depth analysis of the shift intentions of medical students and medical professionals by constructing a theoretical hypothesis model, which can be seen in the following Figure 2. The Structural Equation Models Fit Indicators can be seen in Table 11. The CMIN/DF is 1.423, which is less than 3 and reaches the good standard; the IFI, TLI, and CFI are 0.962, 0.957, and 0.961 respectively, which are greater than 0.9 and reach the good standard; the RMSEA is 0.032, and the RMR is 0.065, which is less than 0.08 and reaches the good standard; it shows that the model has good fitness and can be further analysed.

**Table 11:** Results of model fitting indicators.

Indicator	CMIN/DF	RMR	IFI	TLI	TLI	RMSEA
Common indicators	<3	<0.08	>0.9	>0.9	>0.9	<0.08
Measurement model fit results	1.423	0.065	0.962	0.957	0.961	0.032

The following Table 12 shows that the theoretical hypotheses were significantly tested: both procedural switching costs (PSC, *β* = 0.306, z = 5.049, *p* < 0.05) and relational switching costs (RSC, β = 0.240, z = 3.803, *p* < 0.05) showed a significant positive effect on shift barriers (SB), and the role of the PSC was stronger, suggesting that operational process complexity hinders transfer more than interpersonal cost; habit (HA, β = 0.121, z = 2.064, *p* < 0.05) positively strengthens SB, verifying the inhibitory effect of behavioural inertia on transfer; the attractiveness of alternative choices (AA, β = 0.150, z = 2.726, *p* < 0.05) and personal innovation (PI, β = 0.106, z = 2.272, *p* < 0.05) had a significant positive effect on SI, but PI had a weaker effect, suggesting that external opportunities were the main driver of transfers; social factors (SF, β = −0.147, z = −2.355, *p* < 0.05) and the three types of dissatisfaction (DF, β = −0.208; DE, β = −0.133; DS, β = −0.164, all *p* < 0.05) had a significant negative effect on SI, in which DF had the strongest inhibitory effect, while subjective norms (SN, *p* > 0.05) had no significant effect; finally, shift barriers (SB, β = −0.179, z = −3.596, *p* < 0.05) served as the core mediator variable, which completely verified the conduction mechanism of “perceived cost → barrier reinforcement → behavioural inhibition,” and the overall model was highly compatible with the theoretical framework. The model is highly compatible with the theoretical framework.

**Table 12:** Path analysis results.

Path	Estimate	S.E.	C.R.	P	Estimate
SB ← PSC	0.302	0.060	5.049	***	0.306
SB ← RSC	0.224	0.059	3.803	***	0.24
SB ← HA	0.112	0.054	2.064	0.039	0.121
SI ← PI	0.104	0.046	2.272	0.023	0.106
SI ← SF	−0.17	0.072	−2.355	0.019	−0.147
SI ← AA	0.142	0.052	2.726	0.006	0.15
SI ← SN	0.024	0.059	0.407	0.684	0.027
SI ← DF	−0.178	0.051	−3.523	***	−0.208
SI ← DE	−0.124	0.047	−2.652	0.008	−0.133
SI ← DS	−0.179	0.063	−2.828	0.005	−0.164
SI ← SB	−0.171	0.048	−3.596	***	−0.179

***indicates a significant difference, *p* < 0.001.

### 3.6 Multi-group analysis

To better understand the differences in the shift intention of medical students and medical professionals to rural areas, this study conducted a Multi-group analysis. The Model Fit results are presented in Table 13. There is a significant difference between the coefficients of the two models for medical professionals and medical students (*p* < 0.05).

**Table 13:** Model fit summary.

Model	DF	CMIN	*P*
Structural weights	93	137.113	0.002

The specific differences can be seen in Table 14. The Z-values were calculated for medical professionals and medical students in “HA → SB” “PI→SI”“SF → SI”“AA→SI ““DS → SI,” and “SB → SI” paths were significantly different (Z > 1.96). Multi-cluster analyses showed significant differences in the strength of critical path influence between the medical professionals and medical student groups: the negative influence of social factors (SF) on shift intention (SI) was much stronger for the professional staff (*β* = −0.412) than for the medical students (β = 0.08), while the medical student group’s positive effect of personal innovation (PI) on SI (β = 0.175) was significantly higher than that of medical professionals (β = −0.064); furthermore, medical professionals’ sensitivity to functional dissatisfaction (DF) (β = −0.196) was also higher than that of medical students (β = −0.214) suggesting that occupational experience reinforces social normative constraints and pragmatic orientations, whereas the medical student population relies more on innovative traits to drive transfer decisions.

**Table 14:** Results of the multi-group analysis between medical students and medical professionals.

Path	Medical professionals	Medical students	Z
SB ← PSC	0.319**	0.337**	−0.08
SB ← RSC	0.282**	0.146	1.261
SB ← HA	0.248**	−0.016	2.198
SI ← PI	−0.064	0.175**	−2.652
SI ← SF	−0.412**	0.08	−4.083
SI ← AA	−0.003	0.224**	−2.035
SI ← SN	−0.059	0.063	−0.961
SI ← DF	−0.196*	−0.214**	−0.04
SI ← DE	−0.067	−0.172*	0.996
SI ← DS	0.104	−0.425**	4.132
SI ← SB	−0.3**	−0.074	−2.649

*p < 0.05, **p < 0.01.

## 4 Results

### 4.1 The effect of dissatisfaction on shift intention

The research hypothesis H1 proposes that dissatisfaction with the overall environment of rural healthcare negatively affects the shift intention of healthcare professionals. According to the validity test data, the items measuring functional dissatisfaction (DF), affective dissatisfaction (DE) and social dissatisfaction (DS) showed good reliability and validity. This suggests that these three dissatisfaction dimensions are effective in reflecting healthcare practitioners’ negative perceptions of the rural healthcare environment. Specifically, the AVE values of functional dissatisfaction for medical professionals and medical students were 0.664 and 0.549, respectively, the AVE values of affective dissatisfaction were 0.695 and 0.566, and the AVE values of social dissatisfaction were 0.627 and 0.533, which were within the acceptable range. According to the results of the model test presented in Figure 2, the path coefficient of functional dissatisfaction (DF) on shift intention (SI) is −0.208, indicating that shift intention decreases when functional dissatisfaction increases. According to Figure 2, the corresponding path coefficient of affective dissatisfaction (DE) is −0.133, and the corresponding path coefficient of social dissatisfaction (DS) is −0.164. Overall, functional dissatisfaction (DF), affective dissatisfaction (DE), and social dissatisfaction (DS) have a significant negative impact relationship on medical personnel shift intention, and hypothesis H1 is valid, that is, dissatisfaction has a significant negative impact on medical personnel, the hypothesis H1 is true, that is, dissatisfaction has a significant negative effect on the intention of medical personnel to shift.

### 4.2 The effect of the attractiveness of alternatives on the shift intention of medical personnel

Hypothesis H2 that the attractiveness of alternatives has a positive effect on the shift intention of medical personnel. The results of the validity test showed that the alternatives attractiveness (AA) measure had high reliability (Cronbach’s*α* = 0.912 for medical professionals, Cronbach’s α = 0.866 for medical students) and convergent validity (medical professionals: CR = 0.912, AVE = 0.634; medical students: CR = 0.867, AVE = 0.521). Based on Figure 2, the path coefficient of alternatives attractiveness (AA) on shift intention (SI) was 0.150, showing a positive correlation. Hypothesis H2 is valid, i.e., alternatives attraction has a significant positive effect on medical personnel’s shift intention.

### 4.3 The effect of subjective norms on medical personnel’s shift intention

Hypothesis H3 proposes that subjective norms have a positive effect on medical personnel shift intention. Validity test data showed that the subjective norms (SN) measurement items performed well in terms of reliability (Cronbach’s α = 0.875 for medical professionals and Cronbach’s α = 0.866 for medical students) and validity (medical professionals: CR = 0.876, AVE = 0.638; medical students: CR = 0.867, AVE = 0.620). This suggests that social expectations and pressures (e.g., support from family and friends and policy guidance) can greatly influence the shift intention of medical professionals to rural areas. Based on the path coefficient of subjective norms (SN) is 0.027, indicating that intention to shift increases when subjective norms increase, subjective norms have a positive effect on the shift intention of medical personnel. However, the results were not significant, this shows that although hypothesis H3 is valid, this relationship may not be universal, it depends on certain specific conditions. In particular, the large proportion of medical students and young groups in this study may lead to a weak result. At the same time, the relationship may be mediated by other variables rather than appearing directly.

### 4.4 The effect of shift barriers on medical personnel’s shift intention

Hypothesis H4 proposed that shift barriers have a negative effect on shift intentions of healthcare professionals. The results of the validity test indicated that the items measuring the shift barriers (SB) had good reliability (Cronbach’s*α* = 0.846 for medical professionals and Cronbach’sα = 0.844 for medical students) and convergent validity (medical professionals: CR = 0.847, AVE = 0.649; medical students: CR = 0.844, AVE = 0.644). Based on the path coefficient of shift barriers (SB) on shift intention (SI) was −0.179, suggesting that their shift intention decreased when shift barriers increased. Therefore, hypothesis H4 is valid, that is, shift barriers have a negative effect on shift intention of medical personnel.

### 4.5 The effect of personal innovation on shift intention of medical personnel

Hypothesis H5 proposes that personal innovation has a positive effect on the shift intention of medical personnel. The validity test data showed that the reliability (Cronbach’s α = 0.811 for medical professionals, Cronbach’s α = 0.858 for medical students) and validity (CR = 0.882, AVE = 0.652 for medical professionals, CR = 0.859, AVE = 0.604 for medical students) of the personal innovation (PI) measurement items performed well. Based on the path coefficient of the effect of Personal Innovation Intention (PI) on shift intention is 0.106, showing a positive relationship. Therefore, hypothesis H5 is valid.

### 4.6 Influence of social factors on medical personnel’s intention to shift

Hypothesis H6 proposes that social factors have a positive influence on medical personnel’s shift intention to rural areas, but Amos validation results show a negative correlation between social factors (SF) and SI (r = −0.452, *p* < 0.01), H6 is invalid. The reason may lie in social factors such as social networks, family responsibilities, and urban living environments, which may increase healthcare professionals’ dependence on cities and thereby weaken their intention to work in rural areas. This differs from the traditional assumption that “social support promotes intention,” suggesting that the role of social factors is context-dependent. Policymakers need to create favorable conditions, such as improving rural living environments and providing family support policies to mitigate the negative influence of these factors, especially this study focuses on the stability of policy support in special period (after the epidemic).

## 5 Conclusions and policy implications

Based on the PPM framework, this study has systematically analyzed the key factors influencing medical personnel’s shift intention using the PPM model. The findings reveal that dissatisfaction is the main constraint affecting medical personnel’s intention to shift, specifically in terms of work environment, satisfaction of personal emotional needs, and social needs. In addition, the attractiveness of alternative opportunities and subjective norms play a positive role in promoting medical personnel’s intention to shift, which is particularly crucial in attracting medical personnel to shift to rural areas (21). Reasonable shift intention of medical personnel is of great significance in alleviating the shortage of medical personnel in rural areas and improving the quality and effectiveness of medical services. Therefore, this study puts forward the following policy recommendations, aiming to improve the overall satisfaction of medical personnel with rural medical work, promote the balanced development of urban and rural medical services, and realize the overall improvement of the quality of rural medical services and protection system.

### 5.1 Recommendations for medical students

1. Optimizing internship practice combined with environmental improvement: given the significant impact of medical students’ emotional dissatisfaction on their shift intention emphasis should be placed on improving the internship environment for rural medical students. On the one hand, increase the investment in the infrastructure of rural medical students, update medical equipment, and provide more advanced practice conditions for medical students; on the other hand, strengthen the care for medical students in internship and improve the living environment, such as providing comfortable accommodation. At the same time, increase the professional guidance during the internship period, so that medical students can better combine theoretical knowledge with practical operation in practice, enhance their sense of identity and satisfaction with rural medical work, and reduce the shift intention due to emotional factors.
2. Accurate career planning and promotion guidance: considering medical students’ concern for career development, provide them with accurate career planning services. Personalised career planning programmes are formulated according to medical students’ personal interests, professional strengths and career goals. At the same time, medical students are introduced in advance to the development prospects and promotion opportunities in the rural medical industry, such as the establishment of clear promotion paths and standards in rural medical institutions, so that medical students can see the potential for development in the field of rural health care, and enhance their intention to devote themselves to the cause of rural health care.
3. Targeted economic incentives and publicity and education: In order to attract medical students to internships or jobs in rural medical institutions, special economic incentives are provided, such as the establishment of special scholarships for rural medical care and internship subsidies. In addition, strengthen publicity and education on the importance of rural medical services, through case sharing and field visits, etc., so that medical students can have a deep understanding of the needs and significance of rural medical care, stimulate their sense of social responsibility and sense of mission, and reduce their shift intention from the level of emotions and values.

### 5.2 Suggestions for medical professionals

1. Improve the promotion system and work environment optimization: as the negative effect of medical professionals’ dissatisfaction with the work environment is not significant, but in order to further improve their work motivation and stability, it is still necessary to improve the promotion system. A fair, transparent, and scientific promotion mechanism is established, and promotions are assessed according to the medical professionals’ work performance, professional skills, and contribution. At the same time, continue to improve the working environment, in addition to updating medical equipment, should also optimize the workflow, reduce unnecessary administrative burden, so that medical workers can focus more on medical services, improve their job satisfaction.
2. Strengthen welfare protection and career support: For medical professionals, increase welfare subsidies and provide more career protection measures, such as purchasing commercial insurance and providing subsidies for vocational training, in addition to regular welfare subsidies. In addition, more professional training and learning opportunities are provided for medical professionals to help them continuously improve their professional skills and adapt to the needs of rural medical development. At the same time, psychological support and counselling mechanisms are established to ease the work pressure of medical professionals and enhance their sense of belonging and loyalty to rural medical work.
3. Strengthen social publicity and resource support: increase social attention and recognition of rural medical professionals, publicize the advanced deeds and contributions of rural medical professionals through the media, and create a good social atmosphere. At the same time, the government and society should increase the resource support for rural medical institutions, including human, material and financial resources, to provide better working conditions and development space for medical professionals, to fundamentally improve the effectiveness of the rural medical security system, and reduce the intention of medical professionals to transfer.

Through the implementation of these specific policy recommendations for medical students and medical professionals, the needs of different groups can be better met, a fairer and more efficient medical personnel shift intention mechanism and rural healthcare talent protection system can be constructed, and the balanced development of urban and rural healthcare services can be promoted.

## 6 Discussion and limitations

1. Limitations of geographical representation: The distribution and collection of questionnaires in this study were limited by time and resources, covering only five prefecture-level cities in Zhejiang Province, and relying mainly on snowball sampling as a non-probabilistic method, which resulted in systematic biases in the geographical distribution of the samples and individual characteristics. Since snowball sampling cannot guarantee that each individual has an equal probability of being selected, the results of the study should be interpreted with caution and mainly provide exploratory insights.
2. Limitations of the sample structure: The sample of this study mainly consisted of medical students and medical professionals within 5 years of graduation, and lacked medical professionals with many years of clinical experience. Medical students’ shift intentions are often based on abstract career aspirations, and they have not yet experienced the trade-offs between family responsibilities, mature social networks, and the challenges of urban and rural healthcare realities, so their logic of consideration may differ from that of medical professionals. Therefore, the findings of this study are only applicable to the “shift intentions of medical students and early medical professionals” and are not representative of the healthcare workforce as a whole. In the future, it is necessary to expand the coverage of the sample in terms of geography, position and seniority through multi-stage stratified random sampling, and optimize the design of the questionnaire in order to improve the external validity and credibility of the conclusions.
3. Another important limitation of this study lies in the misalignment between some of the theoretical hypotheses and the empirical results. For example, Hypothesis 3 was not supported, and Hypothesis 6 revealed a significant negative relationship rather than the expected positive one. These discrepancies indicate that certain relationships may be weaker or even reversed in specific contexts, particularly given the characteristics of our sample. While such inconsistencies reduce the extent to which the theoretical model is fully validated, they also provide valuable insights by highlighting the context-dependent nature of the relationships. Future research should further examine these unexpected findings with larger and more diverse samples to clarify whether they are specific to the present study or generalizable across settings, or consider the mediating effect.
4. Insufficient exploration of the mechanisms among variables: Although this study initially identified several key factors affecting the intention of medical personnel to move, the analysis of the interactions among these factors and their underlying mechanisms is still superficial. Future research can combine longitudinal tracking design or mixed research methods to further reveal how the factors reinforce or inhibit each other, so as to portray the internal logic of medical personnel shift intention more comprehensively and provide more targeted evidence-based basis for health human resource policies.

## Data Availability

The raw data supporting the conclusions of this article will be made available by the authors, without undue reservation.
